# Modelling Eurasian beaver foraging habitat and dam suitability, for predicting the location and number of dams throughout catchments in Great Britain

**DOI:** 10.1007/s10344-020-01379-w

**Published:** 2020-05-07

**Authors:** Hugh A. Graham, Alan Puttock, William W. Macfarlane, Joseph M. Wheaton, Jordan T. Gilbert, Róisín Campbell-Palmer, Mark Elliott, Martin J. Gaywood, Karen Anderson, Richard E. Brazier

**Affiliations:** 1 University of Exeter, Devon, UK; 2 Department of Watershed Sciences, Utah State University, Logan, UT USA; 3 Independent Beaver Consultant, Edinburgh, UK; 4 Devon Wildlife Trust, Exeter, Devon UK; 5 Scottish Natural Heritage, Great Glen House, Leachkin Rd, Inverness, UK; 6 Environment and Sustainability Institute, University of Exeter, Exeter, Devon UK

**Keywords:** Eurasian beaver, *Castor fiber*, Beaver dams, Dam capacity, Modelling, Management, Habitat

## Abstract

Eurasian beaver (*Castor fiber*) populations are expanding across Europe. Depending on location, beaver dams bring multiple benefits and/or require management. Using nationally available data, we developed: a Beaver Forage Index (BFI), identifying beaver foraging habitat, and a Beaver Dam Capacity (BDC) model, classifying suitability of river reaches for dam construction, to estimate location and number of dams at catchment scales. Models were executed across three catchments, in Great Britain (GB), containing beaver. An area of 6747 km^2^ was analysed for BFI and 16,739 km of stream for BDC. Field surveys identified 258 km of channel containing beaver activity and 89 dams, providing data to test predictions. Models were evaluated using a categorical binomial Bayesian framework to calculate probability of foraging and dam construction. BFI and BDC models successfully categorised the use of reaches for foraging and damming, with higher scoring reaches being preferred. Highest scoring categories were ca. 31 and 79 times more likely to be used than the lowest for foraging and damming respectively. Zero-inflated negative binomial regression showed that modelled dam capacity was significantly related (*p* = 0.01) to observed damming and was used to predict numbers of dams that may occur. Estimated densities of dams, averaged across each catchment, ranged from 0.4 to 1.6 dams/km, though local densities may be up to 30 dams/km. These models provide fundamental information describing the distribution of beaver foraging habitat, where dams may be constructed and how many may occur. This supports the development of policy and management concerning the reintroduction and recolonisation of beaver.

**Electronic supplementary material:**

The online version of this article (10.1007/s10344-020-01379-w) contains supplementary material, which is available to authorized users.

## Introduction

Beaver reintroduction and recolonisation across Europe provides opportunities for conservation and provision of ecosystem services (de Visscher et al. 2014; Law et al. 2016; Puttock et al. 2017, 2018). However, for the species to coexist with humans, particularly in densely populated and intensively managed landscapes, informed policy and management is required (Auster et al. 2019; Crowley et al. 2017; Gaywood et al. 2015). This should be based on a strong understanding of where beaver are likely to be active, where dam impacts/opportunities occur and how many dams may be expected in a catchment. Such understanding is vital to ensure that benefits that beaver offer be maximised, whilst minimising negative impacts on land and infrastructure. Herein, we provide a modelling framework that contributes to this understanding which describes beaver foraging habitat and river reaches suitable for dam construction.

The Eurasian beaver (*Castor fiber*) was extirpated from mainland Great Britain (GB) approximately 400–600 yBP (Kitchener and Conroy 1997; Manning et al. 2014), and populations were significantly reduced throughout Eurasia as a result of hunting (Halley et al. 2012). The species is now expanding throughout mainland Europe alongside an increasing number of enclosed and free-living populations in Scotland and England. Their ability to significantly modify fresh water habitats through dam building, lodge constructing, tree felling and excavating canals and burrows has earned beavers the title of ecosystem engineer (Gurney and Lawton 1996). Dam construction has a profound effect on the landscape, often forming complex wetlands (Gurnell 1998). Beavers construct dams to (i) increase water depth, reducing predation risk (Gurnell 1998), (ii) access food resources (Campbell-Palmer et al. 2016) and (iii) create deep water for food caches (Campbell-Palmer et al. 2016). Dams are typically built on rivers < 6 m wide and < 0.7 m deep (Hartman and Tornlov 2006). Beaver dams vary in size and structure (see examples in SI.11) depending on purpose, environmental setting, channel geometry, age and hydrological regime.

Riverine and riparian systems across Europe have changed significantly since the Holocene because of agricultural intensification and urbanisation (Brown et al. 2018). This is particularly evident in GB where agriculture and sub/urban areas account for 52.9% and 7.4% of land use respectively (Rowland et al. 2017). Such change has diminished the natural functioning of river systems and contributed to an intensification of flood discharges, soil erosion and diffuse pollution (Bilotta et al. 2010), with concomitant impacts on biodiversity and society. Beaver dams can help restore natural function via (i) attenuation of peak flood flows and extension of lag times by increasing storage capacity and surface roughness (Nyssen et al. 2011; Puttock et al. 2017); (ii) increased drought resilience by maintaining base flow, storing water during dry periods and raising ground water tables (Gibson and Olden 2014); (iii) capturing fine sediment and storing nutrients (de Visscher et al. 2014; Puttock et al. 2018); (iv) aggrading incised channels (Pollock et al. 2014); (v) enhancing channel complexity (John and Klein 2004) and (vi) increasing habitat heterogeneity and biodiversity (Stringer and Gaywood 2016).

Beaver activities can also cause human-wildlife conflict where valuable infrastructure or land use is impacted (Auster et al. 2019; Crowley et al. 2017; Gaywood et al. 2015). Many conflicts can be managed to minimise damage whilst addressing animal welfare considerations and delivering aforementioned benefits (Campbell-Palmer et al. 2016). Understanding where dams are likely to be constructed is therefore important for the effective management of conflicts and benefits, especially with rapidly increasing beaver populations across Europe.

Expanding populations of beaver are known to settle a landscape in a way that approximates the ideal despotic distribution hypothesis (Fustec et al. 2001; Nolet and Rosell 1994), where established populations exclude unsettled individuals, as opposed to the ideal free distribution, where animals can move freely between habitats (Ens et al. 1995; Fretwell and Lucas 1969). This hypothesis assumes that, at low population densities, animals will select optimal habitats but, due to their territorial behaviour, animals will sequentially settle in more marginal habitats as population density increases and the availability of preferred habitats decreases (Fretwell 1972; Fretwell and Lucas 1969). The stage of population expansion will also play a role in habitat choice. Hartman (1996) suggests that the search for a mate may lead to the wider distribution of individuals within a catchment. Spatial scale also plays a role in habitat selection, with both the overall availability of woody resources and their distribution within home ranges determining the suitability of a given habitat (Zwolicki et al. 2019).

Existing models, describing beaver habitat or locations suitable for dams, are available. Many are statistical and derived from field measurement (Barnes and Mallik 1997; Curtis and Jensen 2004; Hartman 1996; Hartman and Tornlov 2006; Howard and Larson 1985; McComb et al. 1990; Pinto et al. 2009), providing the basis for understanding beaver habitat preference. However, these models can prove less effective when extrapolated spatially to landscapes with different characteristics (e.g. from arid shrub-dominated landscapes to boreal forest (Barnes and Mallik 1997)) from where the models were derived (Baldwin 2013; Barnes and Mallik 1997; Cox and Nelson 2008; Howard and Larson 1985; McComb et al. 1990; Suzuki and McComb 1998). Some geographic information system (GIS)-based models have used detailed data inputs not widely available or acquired through rigorous digitising or fieldwork campaigns (John et al. 2010; St-Pierre et al. 2017; Swinnen et al. 2017). Whilst providing accurate and locally valuable information, the application of these models at regional/national scales may be costly or infeasible. Other approaches use coarser resolution spatial data, such as rasterised environmental descriptors > 50m^2^ (South et al. 2000; Stevens et al. 2007), that allow for the development of landscape or national-scale understanding, appropriate to policy, but have limited application for local management due to the coarse spatial resolution of results.

When faced with the task of selecting a modelling framework to understand the distribution and number of beaver dams in Great Britain, three recent modelling frameworks were considered:

(i) A recent and novel approach for identifying areas in a catchment that can support beavers was developed by Dittbrenner et al. (2018) who created a Beaver Intrinsic Potential model based on topographic parameters and discounting contemporary land use cover.
(ii) Stringer et al. (2018) developed a habitat model for Scotland; the most recent for a GB landscape. It locates woodland areas (> 0.5 ha) within 50 m of streams with a gradient < 15% to identify suitable beaver habitat; reaches containing woodland and wider than 6 m are classed as unlikely to be dammed. The authors acknowledge that the spatial distribution of dams is complex and therefore limit their predictions to areas likely and unlikely to be dammed. Additionally, they state that the 15% gradient cut-off used in the habitat model would be improved by a gradual classification rather than an absolute one. Stringer et al. (2018) state that, in most instances, the model effectively identifies suitable territory locations; however, the model occasionally fails to identify signs of beaver activity resulting from dispersing animals or where it occurs in discontinuous habitat containing patchy woodland < 0.5 ha.
(iii) Macfarlane et al. (2017) developed the Beaver Restoration Assessment Tool for North American landscapes to determine the capacity for river systems to support beaver dams. Macfarlane et al. (2017) used a rules-based fuzzy inference system which allows for the uncertainty associated with generalist beaver behaviour. Furthermore, when working across large areas with GIS, the datasets used are often either classifications or less precise than field observation. Therefore, traditional statistics, which require high precision data, can be unsuitable. Fuzzy inference offers a way to deal with this uncertainty in a pragmatic way by taking what has been learned from these high precision studies and applying expert rules to datasets that are naturally of lower precision (Adriaenssens et al. 2004; Fisher 1999). Whilst this Beaver Dam Capacity (BDC) model is valuable, it can predict only the maximum number of dams that can be supported; it does not predict the likely number of dams across a given area. However, BDC was found to predict the suitability for damming as those reaches with higher modelled dam-capacity were preferentially selected for damming over those with lower modelled dam-capacity (Macfarlane et al. 2017). Therefore, whilst Beaver Dam Capacity equates to the maximum number dams a section of stream can support, it can also be considered as a metric for estimating the suitability of a given reach for dam construction.

We therefore chose to develop the modelling framework outlined by Macfarlane et al. (2017), as it addresses the need for a contemporary understanding of dam suitability and utilises a fuzzy-rule system that can account for continuous changes in variables, avoiding stringent/unrealistic rule systems. We also present a new Beaver Forage Index (BFI) model which describes the spatial distribution of beaver foraging habitat and uses this information to inform the BDC model.

Using data from field sign surveys across three distinct GB catchments, where beavers are living wild, we gather empirical data which are used to evaluate the efficacy of the BFI for predicting suitable foraging habitat for beaver and the BDC model for predicting the suitability of reaches for dam construction. Furthermore, we evaluate how modelled BDC relates to observed dam density and estimate the number of expected dams at the catchment-scale.

### In support of policy development and management implementation, this study aims to

1. Develop models to predict the distribution of beaver foraging habitat and damming activity for European landscapes, using nationally available datasets.
2. Compare model results with observed beaver foraging signs and damming activity to validate model predictions.
3. Use model results to predict the number of dams that are likely to occur at a catchment scale.

## Methods

### Site descriptions

**Fig. 1:**
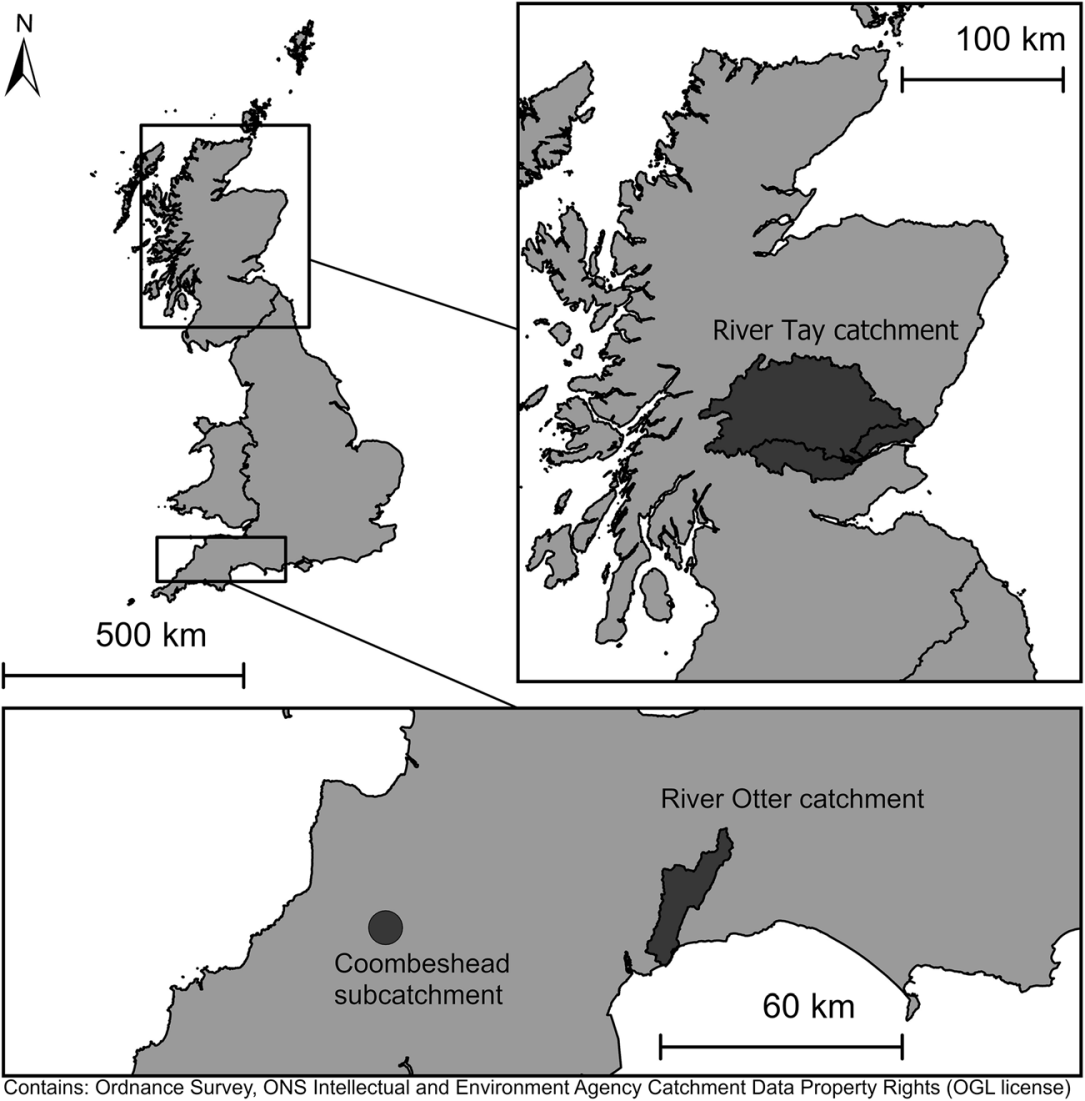
Site locations in GB

Three beaver-impacted sites, representing a range of landscape types, were chosen (Fig. 1). The Tay catchment (including the Earn and Forth sub-catchments), Perthshire has a total area of ca. 6507 km^2^ and ca. 16,139 km of watercourse up to 7th order. Key landcover types comprise arable farming (13%), grazing (39%), urban (2%), coniferous woodland (10%) and semi-natural habitat (30%) (Rowland et al. 2017). Beavers have been living wild in this catchment since at least since 2007 (Gaywood 2017). A catchment-wide survey (Campbell-Palmer et al. 2018 and in review) in 2017 identified 114 active territories.

The River Otter Catchment, Devon is dominated by intensively managed grassland (51%) and arable (29%) farmland, interspersed by patchy areas of semi-natural (11%) and (sub)urban areas (5%) (Rowland et al. 2017). The total catchment area is ca. 250 km^2^ and comprises a total of ca. 595 km of watercourse up to 6th order. Since 2013, when beavers were first publicly reported in the catchment (Crowley et al. 2017), the population has reached approximately 25–40 animals, distributed between eight territories (Brazier et al. 2020).

The third site studied was the Coombeshead subcatchment, within the Tamar catchment in Devon. Free-living beaver family groups have established themselves here and the population has been present since ca. 2015 (Bricknell-Webb 2019 - personal comms). The occupied area comprises 3rd- and 2nd-order streams draining areas of semi-natural woodland (26%), intensively managed grassland (60%), arable land (8%) and heather grassland (5%) (Rowland et al. 2017).

### Producing Beaver Forage Index and Beaver Dam Capacity models

A diagram of the model workflow is shown in Fig. 4 with further details provided in the following subsections.

### Computational requirements

The BHI and BDC models are reliant upon Python 3.6 (Python Software Foundation 2019) and utilises Geopandas (http://geopandas.org/), Rasterio (https://rasterio.readthedocs.io/en/stable/index.html), arcpy (ESRI 2015) and scikit-fuzzy (Warner 2012) modules; code in SI.8. Statistical analysis was carried out using R (version 3.5.1) (R Core Team 2017); code in SI.9. Processing was undertaken on a personal computer with Windows 10 OS, Intel CORE i7 processor and 16GB RAM. Maps were produced using ArcPro GIS version 2.4.1.

### Beaver Forage Index data preparation and execution

Vegetation is important for classifying beaver habitat (Hartman 1996; John et al. 2010; Pinto et al. 2009; St-Pierre et al. 2017). No single dataset contained the detail required to depict all key vegetation types, relevant to beaver foraging. Therefore, a composite dataset was created from OS VectorMap Local data (Ordnance Survey 2018b), The Centre for Ecology and Hydrology (CEH) 2015 land cover map (LCM) (Rowland et al. 2017), Copernicus 2015 20 m tree cover density (TCD) (Copernicus 2017) and the CEH woody linear features framework (WLFF) (Scholefield et al. 2016).

Vegetation datasets were assigned suitability values (zero to five), which are summarised in Table 1. Values were assigned based on a review of relevant literature (Haarberg and Rosell 2006; Jenkins 1979; Nolet et al. 1994; O’Connell et al. 2008), field observation and qualitative comparison with satellite data. Vector data were converted to raster format (resolution of 5 m) to enable array-based calculation between datasets. TCD data were resampled to 5 m (finest common resolution) and aligned with converted vector layers. A full list of suitability values for vegetation datasets can be seen in SI.1–4. An inference system was used to combine these four raster datasets to create a continuous description of the suitability of land cover for beaver foraging at 5 m resolution (Fig. 5). This inference system prioritises the most reliable data for a given land use type; if this dataset contains no value for a given location, the highest value of coincident datasets is used (see SI.8 for BFI code).

**Table 1:** Beaver Forage Index (BFI) value descriptions and the input data land classes attributed to each BFI value for the following data layers: OS Vector, CEH LCM 2015, Copernicus TCD data and CEH WLFF. For further information on all land class values for these datasets see SI.1–4

BFI value	Value description	OS vector classification	CEH LCM 2015 classification	Copernicus tree cover density range (%)	CEH woody linear features framework (WLFF)
0	No vegetation	Boulders, sand, shingle, building, water	Water, rock, saltmarsh, (sub) urban	0	–
1	Unsuitable	Heathland, unimproved grass, marsh	Acid grassland, calcareous grassland, heather, improved grassland, bog	1–3	–
2	Barely suitable	Reeds, shrub and heathland	Arable and horticulture, neutral grassland	4–10	–
3	Moderately suitable	Coniferous woodland, shrub and marsh, shrub and unimproved	Coniferous woodland	11–50	–
4	Suitable	–	–	51–100	WLFF present
5	Preferred	Broad-leafed woodland, shrub, mixed woodland, orchard	Broadleaf woodland	–	–

### Beaver dam capacity data preparation

The stream network (Ordnance Survey 2018) was split into working reaches to extract discrete information following Macfarlane et al. (2017). The network was split at intersections, features < 200 m long were used as final reaches. Features > 200 m were split into the minimum number of equal parts to ensure that all were < 200 m long. Mean reach length across all sites was 122 m (± 47 SD).

The OS Terrain 5 m Digital Terrain Model (DTM) (Ordnance Survey 2017) was stream-burned (Saunders 1999; Turcotte et al. 2001) by reducing the elevation of raster cells coinciding with the vector stream network by 30 m. Elevations at the beginning and end of each reach were extracted, and the difference is divided by the reach length to calculate approximate gradient. Contributing hydrological area for each reach was determined from the intersecting value of a flow accumulation raster layer (Maidment and Morehouse 2002), multiplied by the raster resolution. Reach stream order was determined using the Strahler method (Strahler 1957). As stream order was derived from a burned DTM, post-processing was required; stream order values > 1st order were reduced by one, and erroneous 1st order values, along stream edges, were removed.

To estimate stream power at low and high flows, Macfarlane et al. (2017) used Q2 (high flow) and Q80 (low flow) flow exceedance values, in their North American study. Given the similarity of the structure of dams constructed by *Castor* species, we maintained this standard. Mean daily flow data were obtained (National River Flow Archive 2018) for all gauges within the hydrometric area of each catchment. Q2 and Q80 flow thresholds were calculated for each gauge, and rating curves for flow and contributing catchment area were developed using a non-linear least squares fit. Total stream power at Q2 and Q80 was then calculated for each reach using Eq. 1.

1 $$ \varOmega =\kern0.5em \rho \kern0.5em \mathrm{g}\ Q\ S $$

where *Ω* is stream power (watts/m^2^), *ρ* is water density (1000 kg/m^*3*^), *g* is acceleration due to gravity (9.8 m/s^2^), *Q* is discharge (m^3^/s) and S is slope.

Mean bankfull width was obtained by buffering all reaches to 20 m. Buffers were then clipped by a channel area polygon (Ordnance Survey 2018b). Reach channel area was then divided by reach length to obtain mean width.

Reach BFI values were obtained for two search areas, 10 m (streamside) and 40 m (riparian) from the bank edge. Whilst most beaver foraging takes place within 10 m of a watercourse, feeding can occur > = 40 m from water (Haarberg and Rosell 2006; Iason et al. 2014; McComb et al. 1990). Both search areas extend 100 m up and downstream to account for connectivity of reaches. The mean of the top 50% of BFI values in each search area was extracted to understand the suitability of the best available habitat within a given reach. As beaver can behave as generalists (Nolet and Rosell 1994), they require only limited resources for habitation and dam construction; therefore, this value is more useful for classifying vegetation than the overall mean.

### Beaver Dam Capacity model execution

**Fig. 2:**
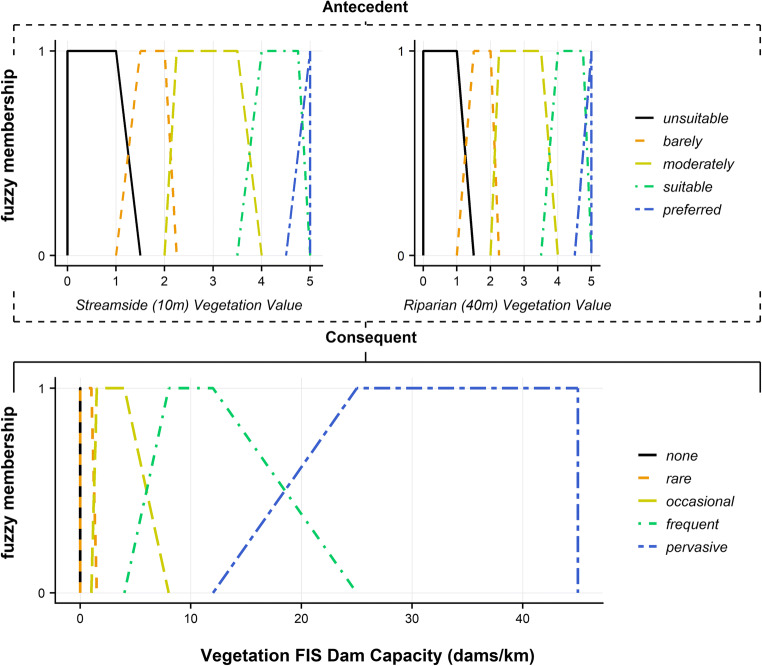
Vegetation fuzzy inference system: antecedent conditions, streamside (10 m) (top-left) and riparian (40 m) vegetation (top-right) suitability are used to derive the consequent dam capacity (base-centre) supported by vegetation

To quantify the number of dams that the habitat within a reach can support, we combined our understanding about the streamside and riparian vegetation suitability. A fuzzy inference system (FIS) (Salski 1992) is used to classify the suitability of surrounding vegetation. The framework for the vegetation FIS is based upon Macfarlane et al. (2017); however, alterations to the rules list (SI.5) and thresholds were incorporated to account for differences in vegetation type, land use and input data in the more intensively managed European landscapes studied herein. Figure 2 shows the FIS design with streamside and riparian BFI values as antecedent variables and dam capacity as the consequent variable.

**Fig. 3:**
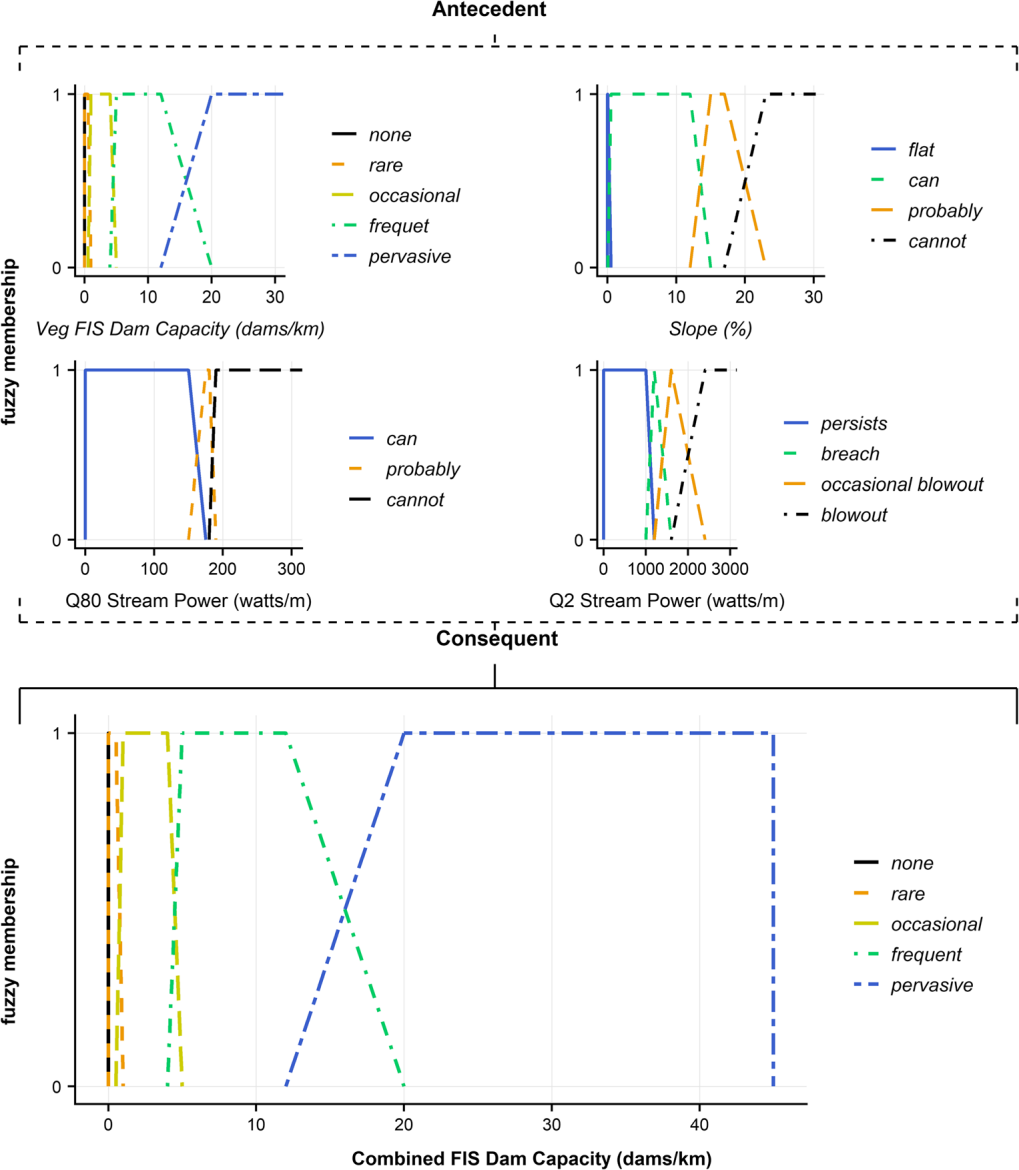
Combined fuzzy inference system design: vegetation dam capacity (top-left), slope (top-right), Q80 (mid-left) and Q2 (mid-right) stream power are antecedents, consequently providing dam capacity (base-centre)

The output from the vegetation FIS, low-flow stream power (Q80), high flow stream power (Q2) and slope are combined using a second FIS. The rules list is presented in SI.6 and Fig. 3 depicts the model mechanism.

**Fig. 4:**
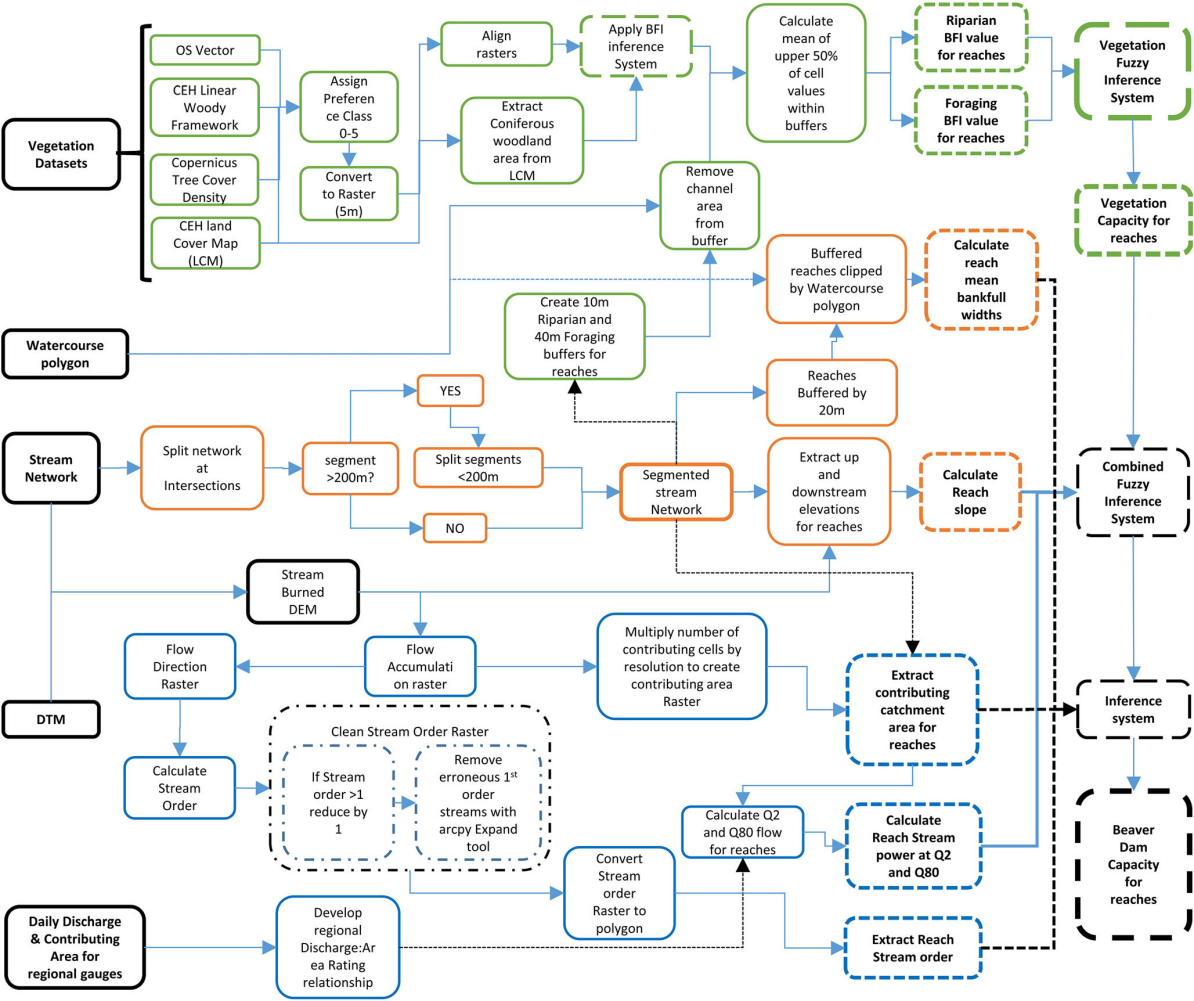
Beaver Dam Capacity (BDC) model workflow. Black (solid outline)—input data, green—vegetation processing, orange—terrain processing, blue—hydrology/hydraulic processing, black (dashed outline)—(fuzzy) inference systems

Following the combined FIS, an inference system was used to constrain the model further. Reaches with an average width > 25 m (to differentiate large waterbodies/lakes), a contributing catchment area > 250 km^2^ or stream order > 5th are considered to have no dam capacity; 5th order streams are capped at 0.9 dams/km; stream orders ≤ 4th remain unchanged. The full modelling workflow is summarised in Fig. 4. These constraints are in-line with other studies (Gurnell 1998; Rosell et al. 2005; Stevens et al. 2007) that observed dam construction very rarely in 5th order streams and never in > 5th order streams.

Following model execution, simplified dam capacity categories were established to facilitate interpretation. Reaches with a capacity of 0 dams/km were categorised as ‘None’, those with 0–1 dams/km as ‘Rare’, 1–4 dams/km as ‘Occasional’, 5–15 dams/km as ‘Frequent”and 16–30 dams/km as ‘Pervasive’.

### Field sign survey

Field surveys were conducted between October 2017 and January 2018. All areas known to contain beaver were surveyed, covering ca. 1310 km (11%) of the River Tay catchment, ca. 61 km (10%) of the River Otter catchment and 1.8 km (29%) of the Coombeshead sub-catchment. Feeding sign locations, observed dams and known locations of removed/collapsed dams were recorded using a handheld Global Navigation Satellite System (Trimble Geo 7X) and loaded into a GIS (ArcPro 2.4.1). Campbell-Palmer et al. (2018 and in review) provide full detail of field-survey protocols.

All feeding sign and dam locations were ‘snapped’ to the nearest reach using Python packages: Shapely (version 1.6.4) and GeoPandas (version 0.6.0). All reaches that intersected a feeding sign or dam were classified as active and or dammed. The number and density of dams per active reach were then calculated (Table 3).

Dams and field signs located within the enclosed site discussed in Law et al. (2016) were excluded from the analysis as it is unclear if the damming observed within enclosures is representative of natural damming behaviour.

### Evaluating Beaver Forage Index

The mean of the top 50% of BFI values, within a 40-m buffer area for each reach, were used to evaluate the efficacy of the BFI index in predicting the suitability of habitat for beaver. The resulting continuous values are derived from a range of integers; to reflect this change, we classified these scores into five categories: unsuitable (< = 1), low (< = 2), medium (< = 3), high (< = 4), preferred (< = 5). Subsequently, we calculated the number of active and non-active reaches within each BFI category. A categorical binomial Bayesian model was then undertaken using the ‘RStan’ package (Stan Development Team 2018) to determine the probability that a reach within a given category may contain signs of beaver activity. An uninformative, uniform prior was used to allow the full range of probability to be objectively explored (Wade 2000). Following the calculation of posterior probability distributions for all BFI categories, the Maximum A Posteriori (MAP) and 95% credible intervals (CI) were derived Fig. 6. Bayes factors (Hooten and Hobbs 2015) were used to quantify the relative likelihood of observing signs of beaver activity between reaches of different categories (Table 1).

### Evaluating Beaver Dam Capacity model results

BDC results were evaluated to determine whether or not BDC was an effective predictor of reaches that were suitable for dam construction. This was carried out, as with BFI, using a binomial Bayesian framework. Once again, a generative binomial model was applied for each of the 5 BDC categories; the MAP and CI were derived from the posterior distribution (Fig. 9) and Bayes factors were derived (Table 2). This Bayesian approach was used to evaluate the results of the BDC and BFI models because it explicitly describes the probability of an outcome (either activity or dam construction) and the uncertainty associated with that outcome (Ellison 2004), allowing us to evaluate precisely the relative preference of beaver towards reaches from the different categories.

**Table 2:** Bayes factor matrix—describing the relative likelihood of observing signs of beaver activity between different BFI categories. Numbers in italic show the MAP Bayes factor, and 95% credible intervals are given in square brackets

Unsuitable (**< =***1*)				
*13.54* [10.78, 17.74]	Low (**< =***2***)**			
*17.97* [14.43, 23.38]	*1.32* [1.15, 1.52]	Moderate **(< =***3***)**		
*18.53* [15.35, 24.99]	*1.41* [1.23, 1.64]	*1.06* [0.94, 1.22]	High (< = *4*)	
*30.71* [25.36, 40.6]	*2.31* [2.05, 2.62]	*1.71* [1.56, 1.95]	*1.64* [1.45, 1.84]	Preferred (< = *5*)

### Predicting numbers of dams

The modelled maximum number of dams per reach was compared with observed dam numbers to determine if modelled dam capacity is an effective predictor of observed dam density. Analysis was carried out for active reaches only to minimise the effect of limited range expansion. Of the 2104 active reaches, 58 contained dams. The distribution was therefore zero-inflated and over-dispersed, as confirmed by the ‘dispersion test’ function from the AER package (Kleiber and Zeileis 2008). A range of models are available for modelling zero-inflated distributions (Martin et al. 2005). Four different zero-inflated models (Hurdle and zero-inflated with both negative binomial and poisson distributions) and two general linear models (poisson and negative binomial distributions) were compared. Performance, fit and over-dispersion of these models were evaluated using Akaike information criterion (Bozdogan 1987), the Vuong test for nested models (Merkle and You 2018) and hanging rootograms (Kleiber and Zeileis 2016). The zero-inflated negative binomial (ZINB) model was selected as it had the best overall performance (Fig. 10a). Dam numbers and 95% confidence intervals (CI) (‘boot’ package, Canty and Ripley 2017) were then calculated for all catchments with the assumption that all reaches were active (Table 3).

**Table 3:** Bayes factor matrix—describing the relative likelihood of beaver dam construction in active reaches of different BDC categories. Numbers in italic show the MAP Bayes factor, and 95% credible intervals are given in square brackets

None				
*20.16* [9.91, 1932.14]	Rare			
*38.06* [17.31, 3395.36]	*1.48* [0.74, 4.01]	Occasional		
*46.14* [23.7, 4517.14]	*1.86* [1.01, 5.25]	*1.16* [0.61, 2.89]	Frequent	
*78.5* [44.23, 7574.58]	*3.54* [1.99, 8.43]	*1.96* [1.2, 4.7]	*1.59* [0.94, 3.34]	Pervasive

To evaluate the ZINB model’s predictive performance, a cross-validation approach was used (Fig. 10b) (Picard and Cook 1984). Data were randomly split into training (70%) and test (30%) subsets 1000 times. The training subset was used to derive a ZINB model. The test dataset was randomly subset at every percentile (100 subsets) to test the model over a range of different scales (ca. 600 m–70 km of channel). The ZINB model was applied to all test data subsets, and the sum of the predicted number of dams for the subset was calculated. A linear regression (with zero-intercept) and prediction intervals were derived for predicted versus observed dam numbers to assist with the assessment of the model performance (see SI.9 for model selection, CI derivation and cross-validation code).

## Results

### Field survey results

Surveys carried out across the three study sites revealed that a total of 2104 reaches (258 km of stream) contained signs of beaver activity (Table 3). As the largest catchment with the most established beaver population, the Tay catchment contains by far the largest length of active river channel (221 km). Thirty-five kilometres of the River Otter and < 2 km of the Coombeshead subcatchment were found to contain signs of beaver activity (Table 3).

A total of 89 dams were identified in 58 different reaches across all catchments with 41, 35 and 13 in the Tay, Otter and Coombeshead (sub)catchments respectively (Table 3).

### Evaluating Beaver Forage Index (BFI) model results

**Fig. 5:**
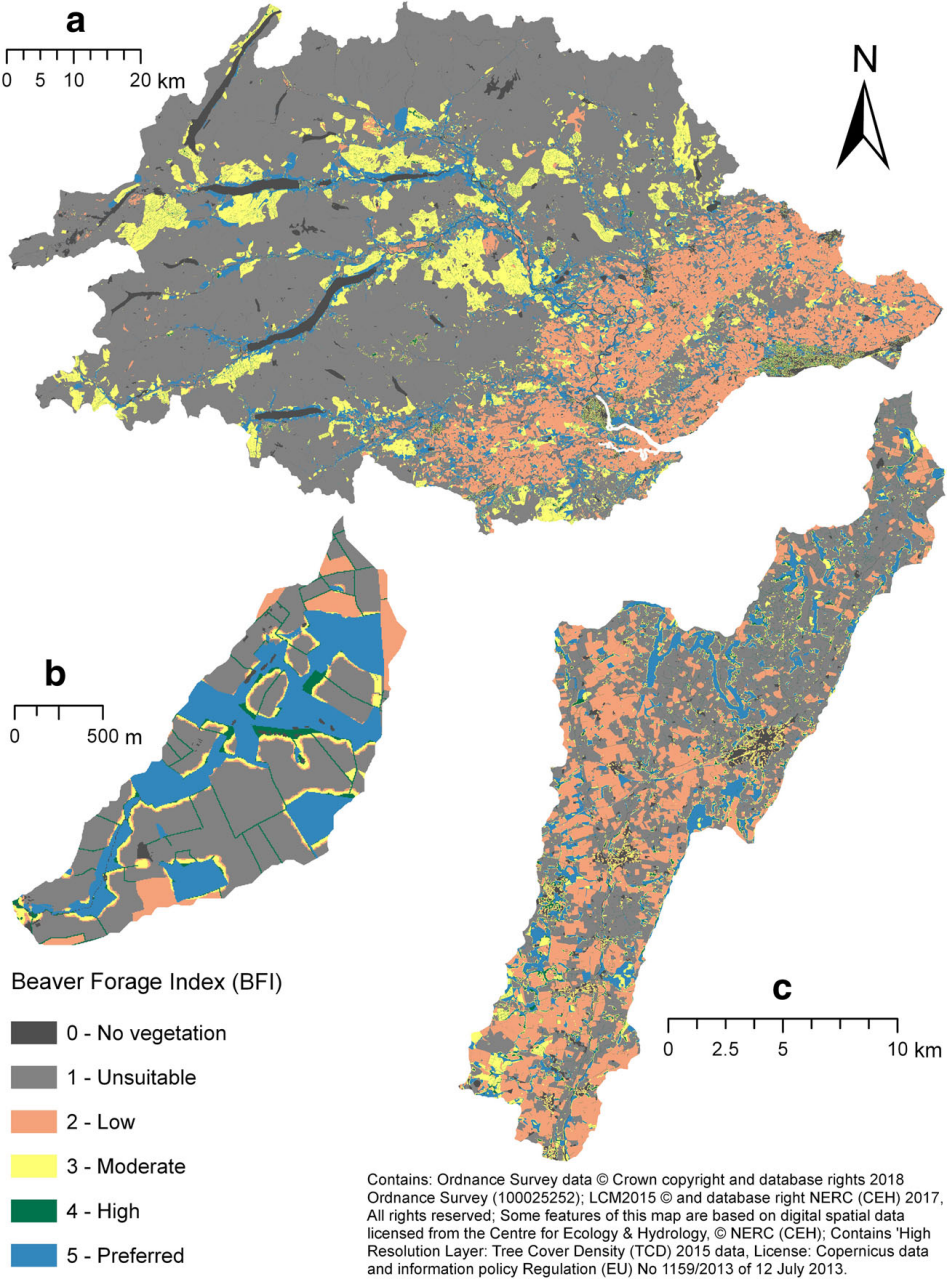
Beaver Forage Index (BFI) model results for **a** R. Tay catchment, **b** Coombeshead subcatchment and **c** R. Otter catchment. The BFI describes the preference of beaver towards a given land cover type, where areas of greater deciduous woodland cover and or suitable forage types are considered preferable

**Fig. 6:**
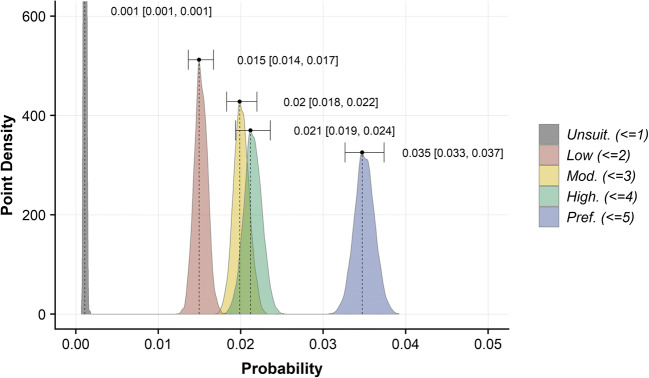
Posterior probability distribution point density plot for Beaver Forage Index (BFI) categories. Maximum A Posteriori (MAP) and 95% credible intervals (in square brackets) are provided for each category

The BFI clearly distinguishes regions of varying landcover; for example, in Tayside, upland areas were markedly devoid of suitable forage; lowland arable agriculture and coniferous woodland provide moderate forage suitability, and riparian deciduous woodland provides the most suitable foraging habitat for beavers (Fig. 5a). Visual inspection of the BFI suggested good levels of coincidence with suitable habitats identified in satellite/aerial data (SI.11). Binomial Bayesian evaluation revealed that, with an increasing BFI value within 40 m of the riverbank, there was a corresponding increase in the probability that a reach would be active (Fig. 6). Preferred reaches, with 40-m BFI scores > 4, were 1.64 (95% CI [1.45, 1.84]) times more likely to be active than those reaches with a score > 3 and < 4. Unsuitable reaches, with a 40-m BFI scores < = 1 were 30.71 (95% CI [25.36, 40.6]) times less likely to be active than preferred reaches. Reaches with scores > 1 and < = 4 (categories: low, medium, high) displayed relatively tightly grouped probabilities of observing signs of beaver activity; medium and high groups were slightly more likely to be active than those in the low category but these groups display clear overlap in credible interval range suggesting these categories have comparable suitability for beaver foraging.

### Evaluating Beaver Dam Capacity model results

**Fig. 7:**
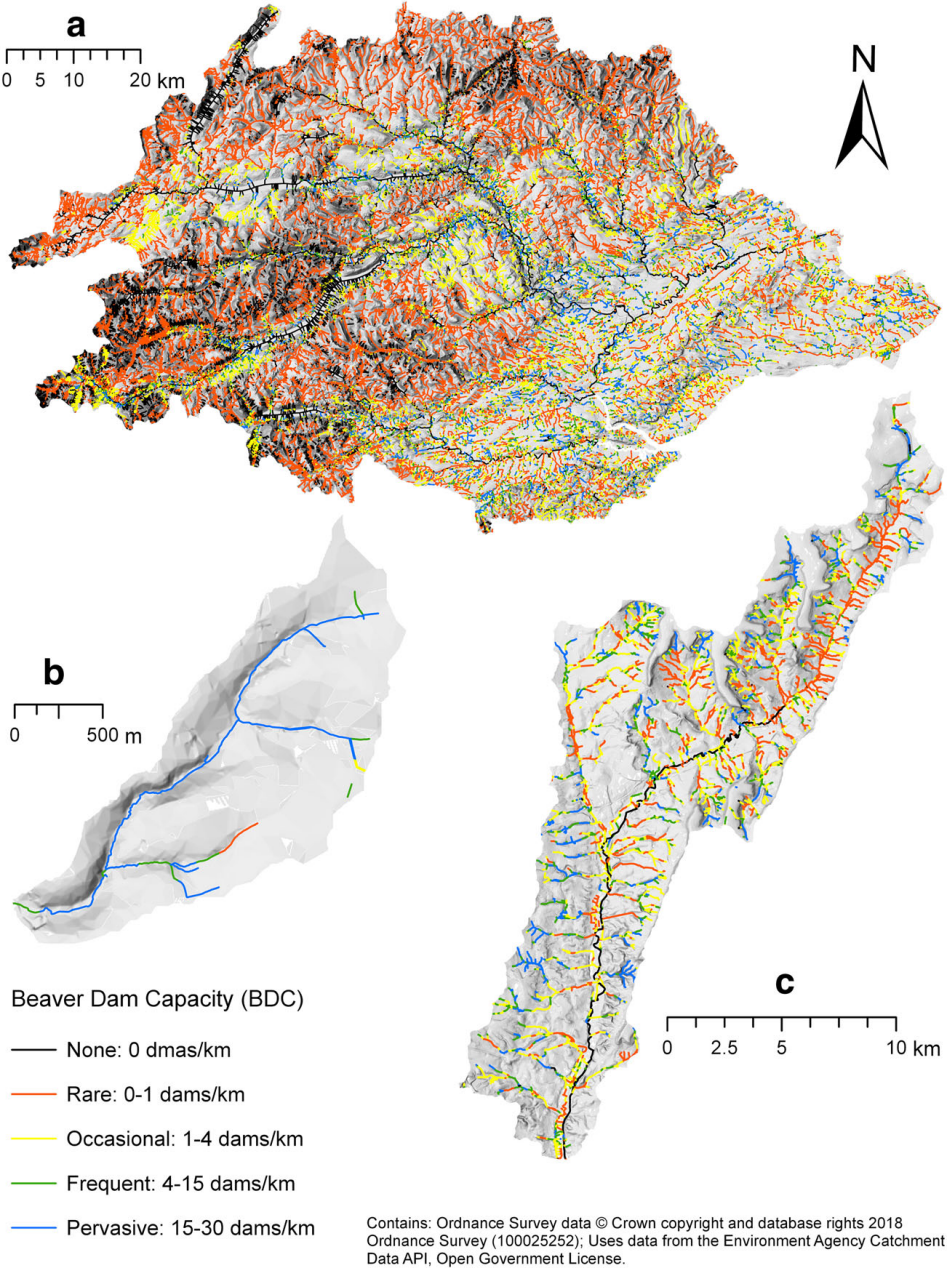
Beaver Dam Capacity (BDC) model results for **a** R. Tay catchment, **b** Coombeshead subcatchment and **c** R. Otter catchment. BDC describes the density of beaver dams that can be supported within a given reach

Broad spatial patterns in the BDC model results can be observed (Fig. 7). For the Tay, the majority of reaches within frequent and pervasive categories are in lowland areas where food and building resources are plentiful, and stream gradients lower (Fig. 7c). Low capacity reaches are common in upland regions where deciduous woodland is lacking, and steeper gradients dominate. In the Otter catchment, areas of higher dam capacity predominate in lower order streams within deciduous woodland (Fig. 7b). Areas of lower capacity were most prevalent within intensively managed grasslands and on larger rivers where stream powers are high.

Much of the Coombeshead subcatchment is classified as pervasive (Fig. 7d). Only reaches with reduced access to woody vegetation had lower capacity. Additional maps illustrating model outputs are provided in SI.11.

**Fig. 8:**
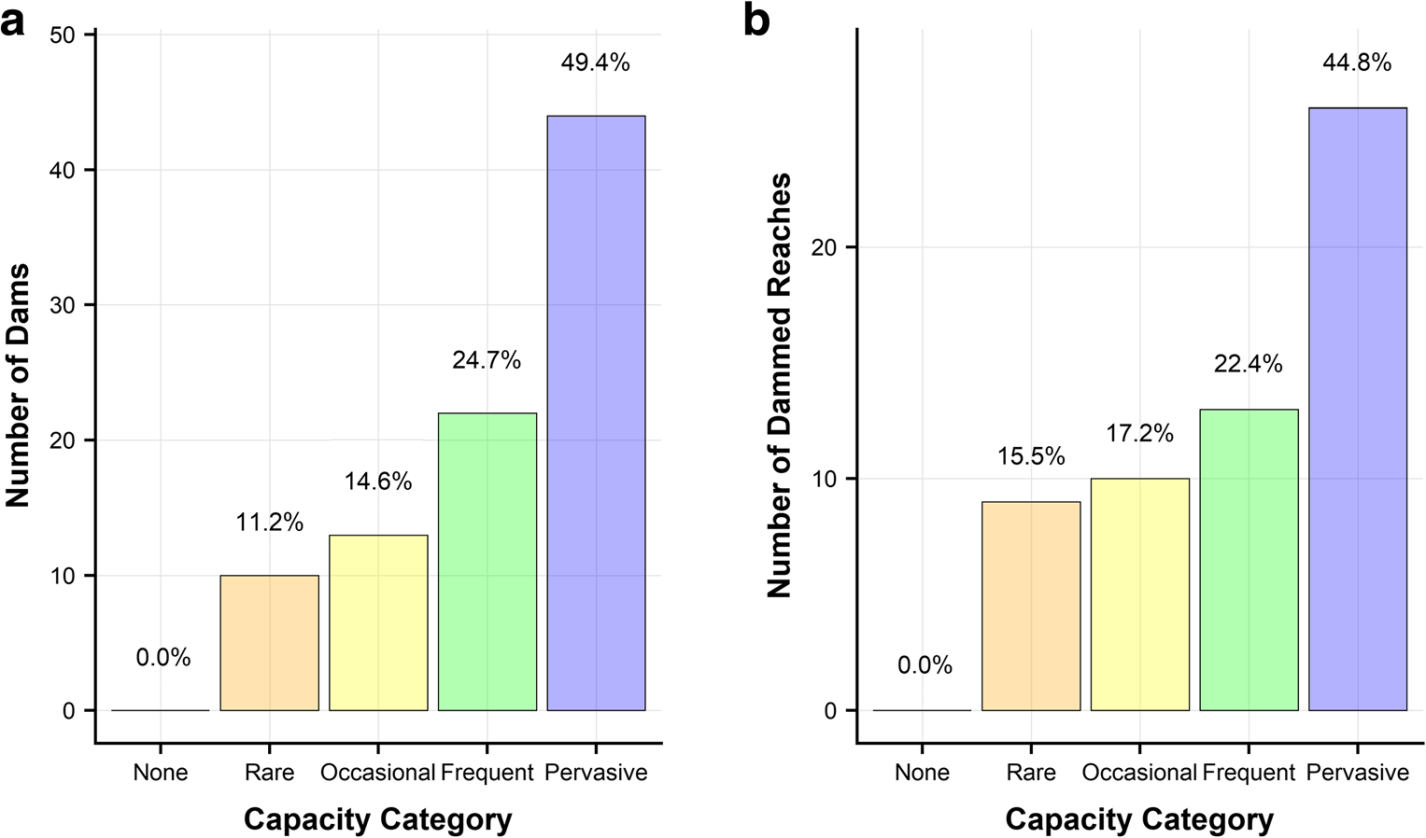
Bar plots showing the number of dams that were observed during field surveys, across all three sites, within different dam capacity categories. **a** Total number of dams per capacity category. **b** Number of dammed reaches within each dam capacity category

Figure 8 shows total number of dams (Fig. 8a) and total number of dammed reaches (Fig. 8b) in each category across all catchments. No dams were observed in reaches where the BDC model predicted no capacity. An increasing number of dammed reaches are observed with higher capacity categories. 74.1% of dams and 67.2% of dammed reaches were observed in pervasive or frequent capacity categories.

**Fig. 9:**
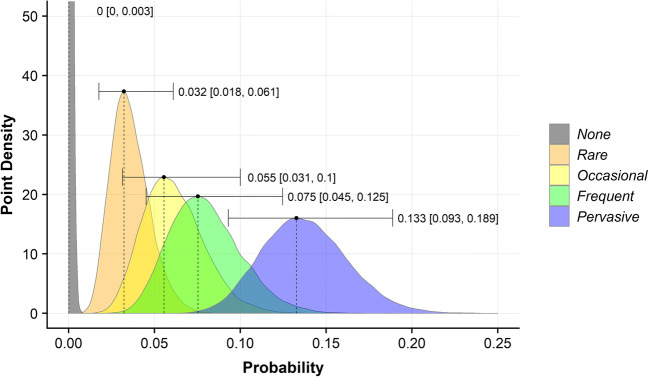
Posterior probability distribution point density plot for Beaver Dam Capacity (BDC) model categories shows the probability of dam construction when a reach is active for all BDC categories. Maximum A Posteriori (MAP) and 95% credible intervals (in square brackets) are provided for each category

Figure 9 shows the posterior probability distribution from the binomial Bayesian analysis of dammed active reaches. With increasing dam capacity, there is a corresponding increase in the probability that a reach will be dammed. Table 2 shows the relative likelihood of dam construction between categories and, for example, shows that active ‘Pervasive’ reaches are 3.54 (95% CI [1.99, 8.43]) times more likely to be dammed than active ‘Rare’ reaches. Notably, the probability of dam construction almost doubles between ‘Frequent’ and ‘Pervasive’ reaches from 0.075 (95% CI [0.045, 0.125]) to 0.133 (95% CI [0.093, 0.189]).

### Predicting numbers of dams

**Fig. 10:**
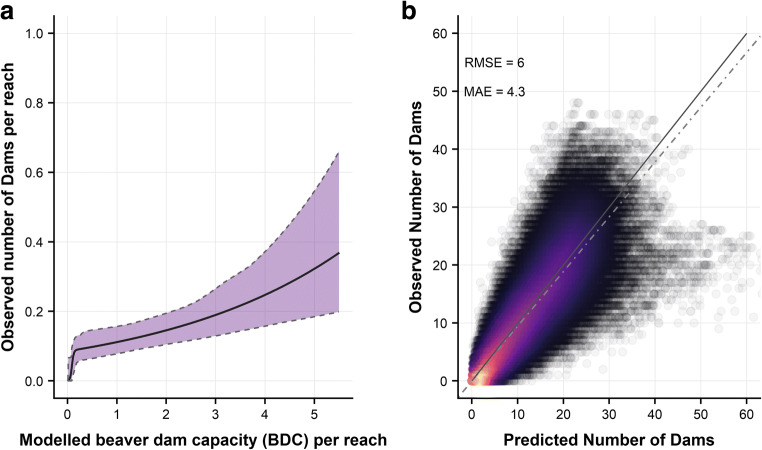
Zero inflated negative binomial (ZINB) regression was used to evaluate the relationship between modelled Beaver Dam Capacity (BDC) and observed numbers of beaver dams for reaches containing beaver activity. **a** The ZINB model, where the coloured zone indicates the 95% confidence bands. **b** Cross validation was used to evaluate the performance of the ZINB model across different scales (600 m–70 km): dashed line shows a linear regression (with zero intercept) and the dotted line indicates the 1:1 line

Modelled dam capacity and observed dam density are significantly related (*p* = 0.013) in the count portion of the ZINB model and in the presence/absence portion (*p* = 0.006) (Fig. 10a and SI 16). This result indicates that BDC is both an effective predictor of dam counts across large spatial scales and for understanding the probability of dam presence or absence for a given reach, aligning with findings from the binomial Bayesian analysis of dam frequency per category in active reaches. Cross-validation showed a strong correlation between the ZINB model prediction and observed dam counts (Fig. 10b). Root mean square error (RMSE) and mean absolute error (MAE) were 6.1 and 4.3 respectively.

Results from the BDC and ZINB models are presented in Table 3. The majority (67.8%) of reaches across the Tay are shown to have no or rare capacity to support dams and only 12.8% are classified as frequent/pervasive. This suggests approximately 51% of the predicted number of dams would be contained within just 12.8% of the river network. Under a scenario where all reaches contain signs of beaver activity, the ZINB model predicts that 6173 (95% CI [3385, 11,597]) dams may be constructed across the whole catchment (ca. 6500 km^2^), equivalent to a density of 0.4 (95% CI [0.2, 0.7]) dams/km. The pervasive category accounts for the largest proportion of predicted dams; however, occasional reaches are predicted to support more than frequent reaches due to the greater length of channel within the category (3142 km and 1030 km for occasional and frequent reaches respectively).

Thirty-six percent of the River Otter was classified as having rare or no capacity to support dams. Fifty-nine percent of the predicted number of dams, if all reaches were active, were predicted to occur within frequent and pervasive reaches which make up 31% of the river network. Reaches classed as occasional are expected to support the second highest proportion of dams (ca. 32%), due to the high prevalence of this reach type (34% of the river network). The predicted number of dams that may be built throughout the catchment (ca. 250 km^2^), under a total occupancy scenario, is 468 (95% CI [262, 814]) or 0.8 (95% CI [0.4, 1.4]) dams/km. A scenario whereby all reaches within a catchment contain signs of beaver activity, at any one time, is highly unlikely; therefore, these figures should be considered an upper estimate of what may be expected if catchments reach population carrying capacity and no management, such as the removal of beaver or dams, is undertaken.

The Coombeshead subcatchment was dominated by reaches with a high capacity to support dams. The predicted number of dams for this subcatchment was between 6 and 16. Currently, there are 13 dams within this subcatchment.

It is also notable that, at present, 64% and 56% of observed beaver signs (by channel length), in the Tay and Otter catchments respectively, are located within reaches with no capacity to support damming (Table 4).

**Table 4:** Results for all sites showing the length of channel, modelled dam capacity, predicted number of dams and number of observed dams

AOI	Capacity category	Channel length (km)	Stream network (%)	Length of active channel (km)	Active channel (%)	Observed dams (*n*)	Observed dams (%)	Predicted *n* dams across catchment (n-dams [95% CI])	Predicted dams (%)
Tay	None	3088.04	19.13	141.69	64.01	0	0	17.4 [0, 81.92]	0.28
	Rare	7838.85	48.57	27.83	12.57	6	14.63	646.91 [122.42, 2284.27]	10.48
	Occasional	3141.86	19.47	14.87	6.72	8	19.51	2335.09 [1315.73, 4181.78]	37.83
	Frequent	1029.69	6.38	17.16	7.75	8	19.51	1162.44 [765.73, 1865.61]	18.83
	Pervasive	1040.73	6.45	19.8	8.95	19	46.34	2011.12 [1181.63, 3184.27]	32.58
	All	16,139.16	100	221.35	100	41	100	6172.95 [3385.51, 11,597.85]	100
Otter	None	33.71	5.67	19.43	56.18	0	0	0.23 [0, 1.09]	0.05
	Rare	178.34	30.02	5.46	15.78	4	11.43	37.5 [6.64, 95.29]	8.01
	Occasional	199.38	33.56	5.69	16.45	5	14.29	150.68 [84.02, 273.91]	32.2
	Frequent	92.95	15.64	2.76	7.99	14	40	106.85 [69.94, 172.75]	22.83
	Pervasive	89.76	15.11	1.24	3.6	12	34.29	172.72 [101.81, 271.09]	36.91
	All	594.14	100	34.58	100	35	100	467.97 [262.41, 814.13]	100
Coombeshead	None	0.01	0.21	0	0	0	0	0 [0, 0]	0.01
	Rare	0.29	4.35	0	0	0	0	0.05 [0.01, 0.12]	0.45
	Occasional	0.23	3.53	0	0	0	0	0.19 [0.11, 0.33]	1.78
	Frequent	0.99	15.1	0	0	0	0	1.06 [0.72, 1.64]	10.16
	Pervasive	5.04	76.81	1.65	100	13	100	9.14 [5.27, 14.28]	87.6
	All	6.57	100	1.65	100	13	100	10.44 [6.11, 16.38]	100

## Discussion and conclusions

In this study, we have developed two models to predict: (i) the spatial distribution of beaver foraging habitat and (ii) the suitability of river reaches for beaver damming. Using empirical survey data, showing the spatial distribution of beaver foraging signs and dam locations, we validated the model predictions across three distinct (sub)catchments. This revealed that models effectively predicted the suitability of both foraging habitat and reaches preferred for dam construction. Model results were then used to predict the likely number of dams that may occur across (sub)catchments, under a scenario in which all reaches contain signs of beaver activity.

### Modelling the distribution of beaver foraging habitat and damming activity in European landscapes

#### Beaver Forage Index (BFI) model

Vorel et al. (2015) describe beaver as a choosy generalist, implying that, in habitats where their preferred woody forage materials (*Salix* spp. and *Populus* spp*.*) are present, beaver will preferentially feed on these species. However, in locations where these species are not available, or populations have occupied these areas, the behaviour of beaver becomes more generalist (Fustec et al. 2001; Nolet and Rosell 1994; Vorel et al. 2015). Therefore, it is reasonable to assume that any model that effectively incorporates willow or poplar-dominant riparian woodland will, to some extent, reasonably predict primary beaver habitat. However, if more marginal habitats are not included in a model, it is highly likely that, as a population expands and beavers are forced to use generalist strategies to exploit marginal habitat, the model will fail to identify these areas of viable habitat. Such an assertion is in line with the dystopic distribution hypothesis (Fretwell 1972; Fretwell and Lucas 1969) that marginal habitats are important for expanding beaver populations and floating individuals (Nolet et al. 1994). This could explain why Stringer et al. (2018) observed that, whilst most beaver territories occurred in areas with substantive deciduous woodland cover, some did not and were therefore not identified as suitable by their model. In the development of the BFI, we have used similar datasets to Stringer et al. (2018) for defining regions of continuous broadleaved woodland; but, in addition, we have included data which describes other sources of forage such as discontinuous shrub, rough grassland, reeds, arable fields and narrow linear woody features such as hedgerows. The value of adding such datasets is highlighted by Fig. 6 which shows clearly that those reaches within the ‘preferred’ category have far greater probability of containing signs of beaver activity and those with sub-optimal resources may still support beaver but are less preferred. Furthermore, those intermediate categories (‘low’, ‘medium’, ‘high’) were found to have a similar probability of containing signs of beaver activity. We can state therefore, that the BFI results align with our own empirical observations and those of other authors (Fustec et al. 2001; Nolet and Rosell 1994; Vorel et al. 2015) that, whilst beaver primarily choose habitat with preferred woody forage, they can occupy reaches with less abundant or alternate resources.

Temporal variability in habitat selection is not explicitly considered in this study, but we know that seasonal selection of habitat changes due to the increased availability of grasses, forbs, macrophytes and crops (Campbell-Palmer et al. 2016; Law et al. 2014; Svendsen 1980). Excluding macrophytes, for which there is no nationally available data, we have, as far as possible, included these types of habitat within the BFI to ensure that year-round habitat is accounted for. A consideration of the effects of temporal variation in forage choice could provide a spatial and temporal understanding of how beaver utilise resources in a river system, advancing our understanding of their population dynamics.

Through the felling of trees, beaver can alter the community and structure of riparian woodland. In so doing, they can, in arid or high latitude/altitude landscapes, consume preferred foraging resources faster than they can regenerate, leading to the succession of less preferred species (Campbell et al. 2005; Fryxell 2001; Rosell et al. 2005). In temperate landscapes, it is suggested that resource consumption is likely to be exceeded by regeneration (at least at the landscape-scale) and therefore resources will not be totally depleted (Nolet et al. 1994). Therefore, when using this modelling approach for areas under more extreme climatic conditions, landcover datasets should be regularly updated to capture beaver-induced impacts on vegetation structure and composition.

#### Beaver Dam Capacity (BDC) model

Macfarlane et al. (2017) were interested in dam capacity to help inform the design of river restoration projects aiming to mimic the behaviour of beaver in support of, for example, salmonid conservation (Bouwes et al. 2016). Such an approach could be of great value with the increasing interest in natural flood management and restoring natural processes (Dadson et al. 2017; Environment_Agency 2017; Iacob et al. 2014; Lane 2017) across Europe. But, from a management perspective, we have found the concept of dam capacity to often be misinterpreted and presumed to represent a likely outcome in the event of beaver occupancy. Our validation approach has allowed us to interpret the BDC results in an alternative manner. The use of a Bayesian validation procedure tells us precisely the probability of dam construction in each capacity category when reaches are active. These probability estimates provide a tangible metric with which to inform management strategies and monitoring programmes. Furthermore, the increase in the likelihood of damming with increasing BDC scores indicates that BDC results do align with empirical observation.

### Using model results to predict the number of dams that are likely to occur at the catchment scale

Given that BDC is a strong predictor of observed dam counts, we have used it to estimate the number of dams that are likely to occur at the catchment scale using ZINB regression. We acknowledge the uncertainty associated with these predictions (Fig. 10); however, this level of uncertainty becomes less problematic when applied to larger areas of interest, so we therefore suggest that this approach is used at the subcatchment scale as a minimum (ca. ≥ 5 km^2^). Whilst the ZINB model was developed only on reaches where beaver were active, we anticipate that, as populations approach carrying capacity, the relationship between estimated dam capacity and observed dam numbers may change. Therefore, this relationship could be revisited as beaver population densities increase.

Frequent and pervasive reaches, predicted to accommodate the highest number of beaver dams, are predominantly found in areas of riparian woodland. These tend to be associated with land use where the risk of conflict is less, although this can vary between specific sites and ownerships. In the Tay and Otter catchments, predicted dam counts were highest in the pervasive category followed by the occasional category. Occasional reaches are typical of agricultural streams lined by discontinuous woody vegetation. These reaches make up a large proportion of both the Otter (30%) and Tay (20%) catchments, and therefore numerous dams will occur within these reaches but, given the lower probability of dam construction, at a lower density than in the reaches with higher modelled capacity.

Our results show that 64% and 56% of active reaches, in the Tay and Otter catchments respectively, have no capacity to support dams. Therefore, it is reasonable to say that, at present, the majority of beaver populations in these catchments will not construct dams. However, it should also be noted that many human-wildlife conflicts that result from beaver activity result from factors other than dam construction. Such activity includes, but is not limited to, tree felling, burrowing and herbivory on crops (Auster et al. 2019; Campbell-Palmer et al. 2016; Crowley et al. 2017; Gaywood et al. 2015). Whilst aspects of this modelling work may help to build understanding on the distribution of such impacts, it cannot be used to explicitly identify where this activity is more likely.

### Conclusion and directions for future research

Herein, we have demonstrated the ability of models to describe the distribution of beaver foraging habitat and where dams are likely to be constructed and how many may occur. Models were validated using the results from a survey of beaver activity signs across the Tay, Otter and Coombeshead (sub)catchments, providing confidence in model results. The predicted number and distribution of beaver dams provide important insight into the current and future impacts of beaver and what the management implications of beaver might be. Model results show that that dams are more likely to occur in low order streams (< = 4th order) with plentiful woody riparian vegetation and less likely to occur in larger rivers with limited riparian woodland. However, agricultural landscapes with patchy riparian woodland may still provide marginal habitat which can support beavers and their dams. The Tay, Otter and Coombeshead (sub)catchments could support a dam density of 0.4 (95% CI [0.2, 0.7]) dams/km, 0.8 (95% CI [0.4, 1.4]) dams/km and 1.6 (95% CI [0.9, 2.5) dams/km respectively, and, at present, more than half of all reaches containing signs of beaver activity, across the three (sub)catchments, are unlikely to be dammed by beaver. The modelling procedures, outlined in this study, provide new and robust insight into beaver foraging habitat suitability, the distribution of beaver dams and the density of dams that could be expected within European landscapes.

These findings support the development of national policy concerning the reintroduction and recolonisation of beaver across native extents as well as informing local and regional management strategies.

In anticipation of the continued expansion of beaver across Europe, impacts on ecosystem and hydrological function require quantification at catchment-scales. Whilst there is a strong and developing understanding of the localised impacts of beaver (e.g. Catalan et al. 2017; Law et al. 2016; Puttock et al. 2017, 2018), few studies (e.g. Bouwes et al. 2016; Johnston and Naiman 1990; Martin et al. 2015) have monitored their landscape-scale effect. Future work on localised beaver impacts may wish to consider upscaling their findings, using a BDC modelling approach, to estimate the landscape-scale effect beaver might have. Further modelling efforts should aim to determine where beaver activity (including damming, tree felling and burrowing) may result in conflicts to allow appropriate mitigation to be put in place, but also to identify where it should be encouraged to maximise benefits.

## Electronic supplementary material

ESM 1 (TXT 150 bytes)
